# *Toxoplasma gondii* Manipulates Expression of Host Long Noncoding RNA during Intracellular Infection

**DOI:** 10.1038/s41598-018-33274-5

**Published:** 2018-10-09

**Authors:** Kayla L. Menard, Breanne E. Haskins, Anthony P. Colombo, Eric Y. Denkers

**Affiliations:** 1 Department of Biology and Center for Evolutionary and Theoretical Immunology, University of New Mexico, Albuquerque, NM 87131 USA; 2 Sandia National Laboratories, Albuquerque, NM 87185 USA

## Abstract

Long noncoding RNA (lncRNA) are non-protein-coding transcripts greater than 200 nucleotides that regulate gene expression. The field of transcriptomics is only beginning to understand the role of lncRNA in host defense. Little is known about the role of lncRNA in the response to infection by intracellular pathogens such as *Toxoplasma gondii*. Using a microarray, we examined the differential expression of 35,923 lncRNAs and 24,881 mRNAs in mouse bone-marrow-derived macrophages during infection with high- and low-virulence *T*. *gondii* strains. We found that 1,522 lncRNA molecules were differentially regulated during infection with the high-virulence Type I strain, versus 528 with the less-virulent Type II strain. Of these lncRNAs, 282 were co-regulated with a nearby or overlapping mRNA–including approximately 60 mRNAs with immune-related functions. We validated the microarray for 4 lncRNAs and 4 mRNAs using qRT-PCR. Using deletion strains of *T*. *gondii*, we found that the secretory kinase ROP16 controls upregulation of lncRNAs Csf1-lnc and Socs2-lnc, demonstrating that the parasite directly manipulates host lncRNA expression. Given the number of regulated lncRNAs and the magnitude of the expression changes, we hypothesize that these molecules constitute both an additional regulatory layer in the host response to infection and a target for manipulation by *T*. *gondii*.

## Introduction

It is estimated that less than 3% of the genome codes for proteins, yet recent data indicate that up to 80% of the genome is actively transcribed as non-translated RNA^1,2^. The largest group of non-translated, non-ribosomal RNA is long noncoding RNA (lncRNA), conventionally defined as non-protein-coding transcripts greater than 200 nucleotides, that together account for up to 68% of the transcriptome^3,4^. Despite the prevalence of lncRNA, functions have been ascribed for only approximately 1%^5^. lncRNAs are widely involved in gene regulation at both the transcriptional and post-transcriptional level. Known functions of lncRNA molecules include transcriptional coactivation, recruitment of chromatin modifiers, miRNA sponge function, regulation of splicing, and mRNA stabilization^6,7^. Very little is understood regarding the role of lncRNA in innate and adaptive immunity. Yet, emerging evidence strongly indicates that this class of regulatory molecules significantly impacts host immunity and the ability to respond to infection^1,4,7^.

Here, we examined the global lncRNA response to *Toxoplasma gondii*. This intracellular protozoan is known for its ability to infect a wide range of species and establish latent infection in tissues of the central nervous system and skeletal muscle^8^. As a primarily asymptomatic pathogen, establishment of latent *T*. *gondii* infection requires a balance between immune-induction to prevent host death and immune-evasion to prevent parasite elimination. Failure to maintain this balance may result in toxoplasmic encephalitis, as can occur in AIDS populations, underscoring the opportunistic nature of this parasitic pathogen^9^. *Toxoplasma* is well known for its ability to initiate Th1 immunity through induction of IL-12 and to induce the activity of counter-regulatory cytokines such as IL-10^10-12^. In macrophages, which along with dendritic cells serve as a major target of *in vivo* infection, *Toxoplasma* triggers both pro-inflammatory and anti-inflammatory pathways^13–16^. Parasite secreted effectors such as ROP16, GRA15, and GRA24 are known to directly stimulate signaling responses in macrophages and other host cell types through activation of signal transducer and activator of transcription (STAT), NFκB, and p38 mitogen-activated protein kinase (MAPK) pathways^17–22^. Maintaining a balance between pro-inflammatory and anti-inflammatory responses is key to the success of *Toxoplasma* as an intracellular parasite, and we hypothesize that lncRNAs are involved in regulating these responses.

In this study, we report the comprehensive survey of host lncRNAs regulated during *Toxoplasma* infection. Using a commercial microarray, we examined the differential gene expression of 35,923 putative lncRNA and 24,881 mRNA species in mouse bone-marrow-derived macrophages (BMDM). We found that 1,902 unique lncRNAs and 1,927 unique mRNAs were regulated by infection with either the high-virulence RH or the less-virulent PTG strain. Of these lncRNAs, 282 were co-regulated with an associated (adjacent or overlapping) protein-coding gene, and approximately 60 lncRNA species were co-regulated with an infection- or immune-related protein-coding gene. Employing qRT-PCR, we validated the results of the microarray for several of these co-regulated, potentially immune-related lncRNA and mRNA species. Using a *T*. *gondii* strain deleted for the ROP16 gene – which encodes a protein controlling STAT activation – we found that this host-directed kinase controls expression of two lncRNAs. Our new findings show that *T*. *gondii* is a strong stimulator of the lncRNA transcriptome in host cells and that the parasite exerts an important influence on this response in a strain-specific manner.

## Results

### Hundreds of putative lncRNAs are differentially regulated during infection with *T. gondii*

**Figure 1:**
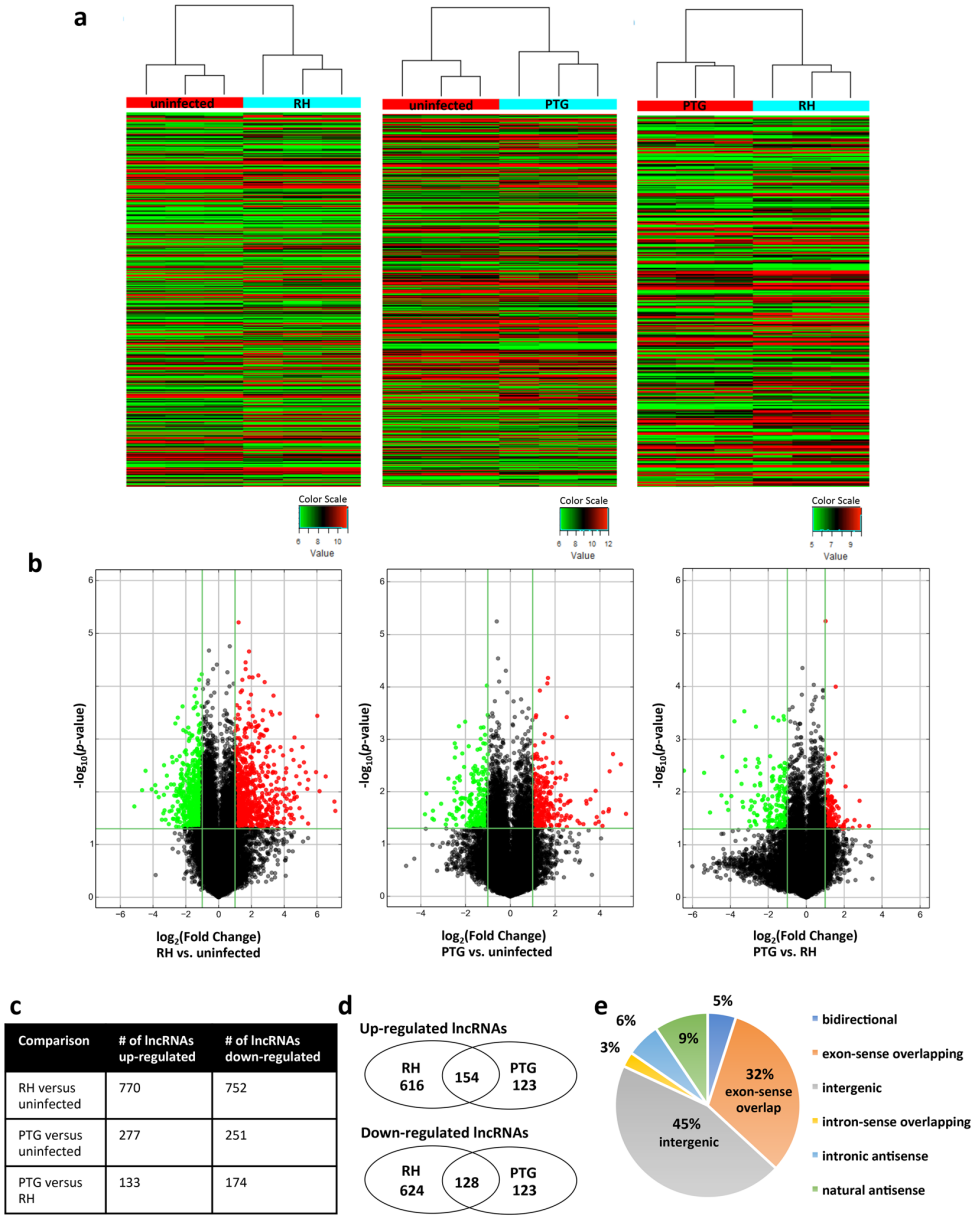
Hundreds of putative lncRNAs are differentially regulated during infection with *Toxoplasma*, as determined using a microarray for mouse lncRNAs. Mouse BMDM were infected with either the highly-virulent RH or the less-virulent PTG strain, and 6 hr later, RNA was isolated for microarray analysis. (**a**) Hierarchical clustering of differentially expressed lncRNAs for uninfected vs. RH-infected, uninfected vs. PTG-infected, and PTG vs. RH-infected. Values in the color scale are normalized intensities. Red bands indicate high relative expression, and green bands indicate low relative expression. (**b**) Volcano plot filtering to visualize fold regulation and statistical significance in lncRNA populations. Statistically significant (*p*-value < 0.05) up (red) or down (green) regulated expression changes (>2-fold) are shown in pairwise comparisons of uninfected, RH-infected, and PTG-infected samples. (**c**) Total number of lncRNA species up- or down-regulated by infection. (**d**) Venn diagrams of up- and down-regulated lncRNA populations reveal shared and unique expression patterns. (**e**) Classification of lncRNA differentially regulated by *Toxoplasma* infection. Experiments were performed in triplicate with BMDM from three separate mice.

We infected mouse bone marrow-derived macrophages (BMDM) with high (Type I, RH) and low (Type II, PTG) virulence *T*. *gondii* at a 4:1 ratio of parasites to cells. This MOI resulted in approximately 80% infection for both strains (Supplementary Fig. S1). Samples of RNA were prepared 6 hr after infection, a time prior to the first parasite mitotic division when on average there was a single parasite per infected cell (Supplementary Fig. S1). Using a commercial microarray (Arraystar), we examined the differential gene expression of 35,923 putative lncRNAs after infection. The array was constructed using public transcriptome databases (Refseq, UCSC knowngenes, Ensembl, etc.), as well as relevant publications^23,24^. Differentially expressed lncRNAs and mRNAs were identified through *p*-value (<0.05) and fold change filtering (>2 fold up or down). The complete dataset is provided in Supplementary Data S1. Hierarchical clustering of the differentially regulated lncRNAs in three replicate experiments indicated that both RH and PTG infections regulated lncRNA expression and that the lncRNA landscape differed between the two infections (Fig. 1a). We also used volcano plot analysis to visualize the differentially expressed lncRNAs. This technique reveals significant changes in large sets of replicate data by plotting fold changes versus *p*-values. We identified substantial populations of lncRNAs that were up- or down-regulated in a statistically significant manner by RH and PTG infection (Fig. 1b). As shown in Fig. 1c, 770 lncRNAs were upregulated during RH infection, and a similar number were down-regulated. Infection with PTG had less extensive effects on lncRNA expression, with a total of 277 species upregulated and 251 down-regulated. 133 lncRNAs were upregulated by PTG relative to RH, while 174 lncRNAs were down-regulated. A total of 282 lncRNAs were either up- or down-regulated during infection with each strain type as depicated by Venn diagrams (Fig. 1d and Supplementary Data S2). However, a substantial number of lncRNAs were regulated in a parasite-strain-specific manner. Taking together all three comparisons (RH vs. uninfected, PTG vs. uninfected, and PTG vs. RH), 1,902 unique lncRNAs were identified in the microarray as significantly differentially regulated. We examined how these lncRNAs were distributed according to protein coding genes (Fig. 1e). The majority of the lncRNAs were either intergenic (>1 kb from the promoter of a protein coding gene) or exon-sense overlapping (overlapping an exon of a protein-coding gene in the sense direction).

Not only were many lncRNAs differentially regulated, but also the magnitude of the observed fold changes was large (Table 1). For all three strain comparisons, the top 20 hits had fold changes larger than ±7, and 31 lncRNAs had fold changes larger than ±20. Notably, infection with high-virulence RH had greater impact on the number and magnitude of lncRNA responses than low-virulence PTG. For example, the average magnitiude of upregulated responses shown in Table 1 was 54.8 and 18.7 for RH and PTG, respectively. These effects could not be attributed to differences in replication rate (and therefore antigen load per cell) because the analyses were carried out 6 hr after infection, prior to the first parasite cell division. Also, the difference in the number of lncRNAs regulated between RH and PTG was not due to invasion efficiency, as percent infection was approximately 80% for each strain.

**Table 1:** lncRNAs with the 20 largest fold changes for each comparison: RH vs. uninfected, PTG vs. uninfected, and PTG vs. RH.

Fold Change	Sequence name	Relationship	Associated gene
**RH versus uninfected**			
139.03	humanlincRNA2313+	intergenic	
133.80	ENSMUST00000143128	exon sense	Chia1
92.56	uc029scs.1	exon sense	Fam169a
71.36	ENSMUST00000176310	exon sense	Six1
64.97	uc007gwf.2	exon sense	Socs2
61.44	AK052777	intergenic	
44.28	ENSMUST00000174768	intergenic	
44.10	ENSMUST00000138295	intergenic	
42.63	TCONS_00036195	intergenic	
39.92	uc008nps.1	exon sense	Tgm2
36.88	ENSMUST00000143423	exon sense	Il1rn
−35.35	AK152734	intergenic	
34.86	AK079750	intergenic	
34.23	ENSMUST00000149686	exon sense	Nup62-il4i1
30.66	AK018095	intergenic	
30.60	AK076251	natural antisense	Hs3st3b1
30.55	AK042611	natural antisense	Ano4
27.28	ENSMUST00000125412	intergenic	
26.73	NR_033499	intergenic	
−25.41	ENSMUST00000060680	intergenic	
**PTG versus uninfected**			
36.04	ENSMUST00000180991	intergenic	
30.72	AK018095	intergenic	
24.10	AK078961	intergenic	
21.71	AK020242	intergenic	
21.24	ENSMUST00000176310	exon sense	Six1
20.94	DT905422	intergenic	
17.81	uc007pnu.1	intronic antisense	Inhba
17.40	ENSMUST00000143423	exon sense	Il1rn
15.50	humanlincRNA2313+	intergenic	
14.58	ENSMUST00000143128	exon sense	Chia1
14.06	uc008nps.1	exon sense	Tgm2
−14.02	uc029skf.1	intergenic	
13.98	ENSMUST00000177234	exon sense	Cav1
−13.44	AK157381	intronic antisense	Rapgef6
11.98	NR_040343	intronic antisense	Crtc3
−11.14	uc009lws.1	exon sense	Lpl
−10.96	ENSMUST00000060680	intergenic	
10.63	ENSMUST00000074412	intron sense	Agpat4
−10.63	ENSMUST00000145263	natural antisense	Ctnnbip1
10.52	uc009luk.1	intergenic	
**PTG versus RH**			
−87.44	uc029scs.1	exon sense	Fam169a
−41.83	ENSMUST00000174768	intergenic	
−33.69	uc007gwf.2	exon sense	Socs2
−22.23	AK086192	intergenic	
−21.47	NR_033499	intergenic	
−18.36	ENSMUST00000165968	intergenic	
−17.34	AK076251	natural antisense	Hs3st3b1
−15.48	ENSMUST00000161131	exon sense	Spag16
−13.81	uc007mlf.1	natural antisense	Sphk1
−12.66	ENSMUST00000155850	exon sense	Hhip
−10.66	AK089320	intergenic	
−10.28	uc007eyw.1	intergenic	
−10.02	ENSMUST00000149686	exon sense	Nup62-il4i1
9.87	uc011wwu.1	exon sense	Pyhin1
−9.61	uc007ecc.1	exon sense	Batf3
−9.25	AK086022	intergenic	
−9.25	AK086961	intergenic	
−8.02	NR_040271	intergenic	
−7.74	AK136179	intergenic	
−7.61	AK035470	natural antisense	Six1

If applicable, associated mRNAs and their relationship to the lncRNA are shown in Columns 3 and 4.

We also note that 17 putative lncRNAs that were regulated by infection mapped to ultraconserved regions of the genome^25^, suggesting that they may function across species (Table 2). This result is potentially significant because lncRNA are generally regarded as poorly conserved through evolution. Using qRT-PCR, we validated expression for one ultraconserved region uc.70 (Supplementary Fig. S2). We note that these ultraconserved regions were identified through genomic sequence comparisons and are generally not based on transcriptomic data. Therefore, the transcripts for these ultraconserved regions would need to be mapped out before further study.

**Table 2:** Differentially regulated lncRNAs that map to ultraconserved genomic regions.

Fold Change	Gene Symbol	Relationship	Associated gene name	Co-regulated
11.2	uc.170	intron sense	Fam172a	No
8.79	uc.47	intergenic		
6.31	uc.462	intronic antisense	Pola1	Yes
5.76	uc.113	intergenic		
5.37	uc.84	intergenic		
3.93	uc.340	intergenic		
3.85	uc.3	intron sense	Casz1	No
3.52	uc.412	intron sense	Aatf	No
2.26	uc.50	intron sense	Srsf7	Yes
2.09	uc.419	exon sense	Srsf1	Yes
−4.00	uc.70	intron sense	Arhgap15	Yes
−3.63	uc.239	intronic antisense	Tox	No
2.84	uc.294	intergenic		
−8.51	uc.275	intronic antisense	Pbx3	No
−7.14	uc.402	intronic antisense	Rpgrip1l	No
−3.07	uc.30	intergenic		
−2.08	uc.473	exon sense	Nlgn3	No

The “Relationship” column denotes the relationship between the lncRNA and its associated (overlapping or nearby) gene. The “Co-regulated” column denotes differential regulation of both the lncRNA and its associated mRNA.

### Hundreds of mRNAs are differentially regulated during infection with *Toxoplasma*

**Figure 2:**
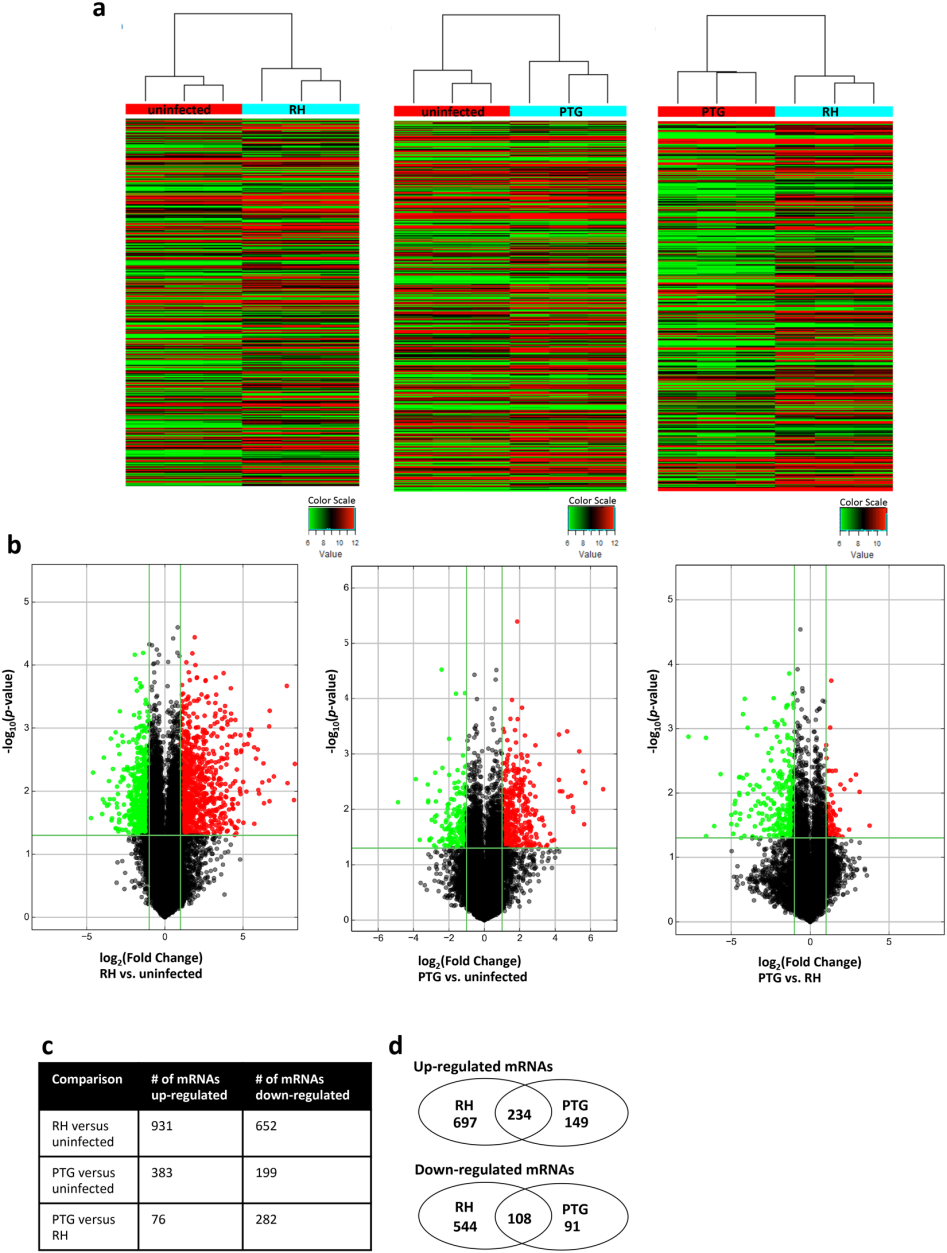
Differential expression patterns of BMDM mRNA during infection with *Toxoplasma*. Changes in mRNA expression were assessed 6 hr after infection of BMDM. (**a**) Hierarchical clustering of pairwise combinations of uninfected, RH-infected, and PTG-infected samples. Values in the color scale are normalized intensities. Red bands indicate high relative expression, and green bands indicate low relative expression. (**b**) Volcano plot filtering to reveal statistically significantly (*p*-value < 0.05) up (red) or down (green) regulated (>2-fold) expression changes in pairwise comparisons of uninfected, RH-infected, and PTG-infected samples. (**c**) Total number of mRNAs up- or down-regulated by infection. (**d**) Venn diagrams of up- and down-regulated mRNA expression reveal unique and shared mRNA species regulated by RH and PTG infection. Experiments were performed in triplicate with BMDM from three separate mice.

In addition to lncRNAs, the microarray contains probes for 24,881 mRNAs, enabling the measurement of changes in expression of protein-encoding transcripts in response to infection. Consistent with results from Fig. 1, hierarchical clustering (Fig. 2a) and volcano plot filtering (Fig. 2b) revealed parasite-strain-specific control of mRNA expression. 1,583 mRNAs were differentially regulated during infection with the high-virulence RH strain compared to an uninfected control (Fig. 2c; the complete data set is shown in Supplementary Data S3). 582 mRNAs were differentially expressed during infection with the less-virulent PTG strain compared to an uninfected control. 358 mRNAs were differentially regulated between RH and PTG. Venn diagrams (Fig. 2d) show that there is overlap (342 total) in the up- or down-regulated mRNAs by RH and PTG infection. A compiled list of mRNAs regulated during both RH and PTG infection is included in Supplementary Data S4. In addition, and particularly for RH, there was substantial parasite-strain-specific regulation of mRNA expression. Altogether, 1,927 unique mRNAs were identified in the microarray as significantly differentially regulated across all three comparisons (RH vs. uninfected, PTG vs. uninfected, and PTG vs. RH). Table 3 shows the top 20 differentially regulated mRNAs for pairwise comparisons of RH vs. uninfected, PTG vs. uninfected, and PTG vs. RH.

**Table 3:** Magnitude of the 20 largest mRNA fold changes during RH and PTG infection in each indicated comparison: RH vs. uninfected, PTG vs. uninfected, and PTG vs. RH.

Fold Change	Sequence Name	Gene Symbol	Description
**RH versus uninfected**			
318.78	NM_011332	Ccl17	chemokine (C-C motif) ligand 17
306.46	NM_020596	Egr4	early growth response 4
230.38	NM_178591	Nrg1	neuregulin 1
223.03	NM_019577	Ccl24	chemokine (C-C motif) ligand 24
125.54	NM_001077508	Tnfrsf9	tumor necrosis factor receptor superfamily, member 9
104.53	NM_009504	Vdr	vitamin D receptor
103.57	NM_001044740	Slc7a2	solute carrier family 7 (cationic amino acid transporter, y^+^ system), member 2
102.04	NM_007759	Crabp2	cellular retinoic acid binding protein II
101.10	NM_130905	Cd209e	CD209e antigen
81.07	NM_010276	Gem	GTP binding protein (gene overexpressed in skeletal muscle)
79.04	NM_008479	Lag3	lymphocyte-activation gene 3
75.30	NM_009398	Tnfaip6	tumor necrosis factor alpha induced protein 6
65.23	NM_001101443	Prrt4	proline-rich transmembrane protein 4
63.07	NM_009864	Cdh1	cadherin 1
61.60	NM_138648	Olr1	oxidized low density lipoprotein (lectin-like) receptor 1
56.87	NM_010215	Il4i1	interleukin 4 induced 1
52.70	NM_146174	Fam115c	family with sequence similarity 115, member C
52.47	NM_008125	Gjb2	gap junction protein, beta 2
45.92	NM_001167996	1110032F04Rik	RIKEN cDNA 1110032F04 gene
42.19	NM_001033248	Gm266	predicted gene 266
**PTG versus uninfected**			
104.88	NM_178591	Nrg1	neuregulin 1
52.15	NM_011851	Nt5e	5′ nucleotidase, ecto
49.93	NM_010809	Mmp3	matrix metallopeptidase 3
47.65	NM_019389	Vcan	versican
41.21	NM_001081249	Vcan	versican
32.62	NM_001080815	Gipr	gastric inhibitory polypeptide receptor
32.09	NM_008361	Il1b	interleukin 1 beta
29.95	NM_001172160	Flrt3	fibronectin leucine rich transmembrane protein 3
−29.38	NM_146511	Olfr107	olfactory receptor 107
26.51	NM_172955	Vcan	versican
25.67	NM_001134475	Vcan	versican
21.13	NM_001134474	Vcan	versican
19.33	NM_001033228	Itga1	integrin alpha 1
18.63	NM_009705	Arg2	arginase type II
18.62	NM_133219	Gcnt2	glucosaminyl (N-acetyl) transferase 2, I-branching enzyme
15.44	NM_001167996	1110032F04Rik	RIKEN cDNA 1110032F04 gene
−14.62	NM_027363	Chp2	calcineurin-like EF hand protein 2
14.01	NM_009398	Tnfaip6	tumor necrosis factor alpha induced protein 6
13.14	NM_212444	Gyk	glycerol kinase
−12.57	NM_001113481	Sema4f	sema domain, immunoglobulin domain (Ig), TM domain, and short cytoplasmic domain
**PTG versus RH**			
208.03	NM_011332	Ccl17	chemokine (C-C motif) ligand 17
96.40	NM_007759	Crabp2	cellular retinoic acid binding protein II
95.86	NM_020596	Egr4	early growth response 4
67.36	NM_146174	Fam115c	family with sequence similarity 115, member C
50.95	NM_008125	Gjb2	gap junction protein, beta 2
31.91	NM_175178	Aifm3	apoptosis-inducing factor, mitochondrion-associated 3
31.78	NM_019577	Ccl24	chemokine (C-C motif) ligand 24
29.07	NM_010415	Hbegf	heparin-binding EGF-like growth factor
27.59	NM_008479	Lag3	lymphocyte-activation gene 3
26.73	NM_001045526	Scimp	SLP adaptor and CSK interacting membrane protein
26.63	NM_175122	Rab39b	RAB39B, member RAS oncogene family
24.98	NM_009864	Cdh1	cadherin 1
22.72	NM_010296	Gli1	GLI-Kruppel family member GLI1
22.59	NM_009252	Serpina3n	serine (or cysteine) peptidase inhibitor, clade A, member 3N
22.20	NM_001101483	Slc35g2	solute carrier family 35, member G2
21.82	NM_145829	Nags	N-acetylglutamate synthase
21.50	NM_130905	Cd209e	CD209e antigen
21.36	NM_001199940	Serpina3i	serine (or cysteine) peptidase inhibitor, clade A, member 3I
20.77	NM_001101443	Prrt4	proline-rich transmembrane protein 4
19.12	NM_001033248	Gm266	predicted gene 266

Gene ontology (GO) analysis identified many immune-related biological processes as differentially regulated in the microarray, including regulation of T-helper cell differentiation, interleukin 2 production, macrophage differentiation, JAK-STAT cascade, and cytokine secretion (Supplementary Fig. S3). Several of the biological pathways identified were regulated in a parasite strain-specific manner. For example, infection with RH was associated with regulation of macrophage differentiation and negative regulation of the JAK-STAT cascade (Supplementary Fig. S3e and f). KEGG Pathway analysis identified many immune-related and infection pathways as differentially regulated in the microarray, including cytokine-cytokine receptor interaction, TGF-beta signaling pathway, Toll-like receptor signaling pathway, RIG-I-like receptor signaling pathway, JAK-STAT signaling pathway, chemokine signaling pathway, Wnt signaling pathway, RapI signaling pathway, measles, malaria, HTLV-1 infection, Hepatitis B, Hepatitis C, and tuberculosis (Supplementary Fig. S4). KEGG analysis also revealed parasite strain-specific pathway enrichment. For example, RH infection was associated with selective enrichment of pathways involved in cytokine-receptor interaction, the JAK-STAT pathway and the p53 signaling pathway (Supplementary Fig. S4e and f).

### Four co-regulated lncRNAs and mRNAs were validated by qRT-PCR

Many known lncRNAs regulate adjacent or overlapping protein-coding genes in a cis-regulatory manner^26,27^. For this reason, we examined the 282 lncRNAs that were co-regulated with a nearby protein-coding gene (Supplementary Data S5). Of these 282, we identified ~60 lncRNAs that were co-regulated with infection- or immune-related protein-coding genes (Supplementary Data S5), suggesting a potential regulatory role for these lncRNAs in the immune response. From this list, we chose 4 lncRNAs to validate by qRT-PCR: Socs2-lnc, Csf1-lnc, Il1rn-lnc, and Ifi44-lnc. Socs2-lnc (uc007gwf.2) is associated with the protein-coding gene Socs2, a suppressor of cytokine synthesis possibly involved in the anti-inflammatory response during *T*. *gondii* infection^28^. Csf1-lnc (ENSMUST00000155557) is associated with the protein-coding gene csf1, a cytokine that drives stem cell differentiation into the macrophage lineage^29^. Ifi44-lnc (ENSMUST00000133888) is associated with Ifi44, an interferon-alfa inducible protein connected with viral infection^30^. Il1rn-lnc (ENSMUST00000143423) is associated with Il1rn, an antagonist cytokine that inhibits activities of interleukin 1 and modulates the interleukin 1 immune and inflammatory responses^31^.

**Figure 3:**
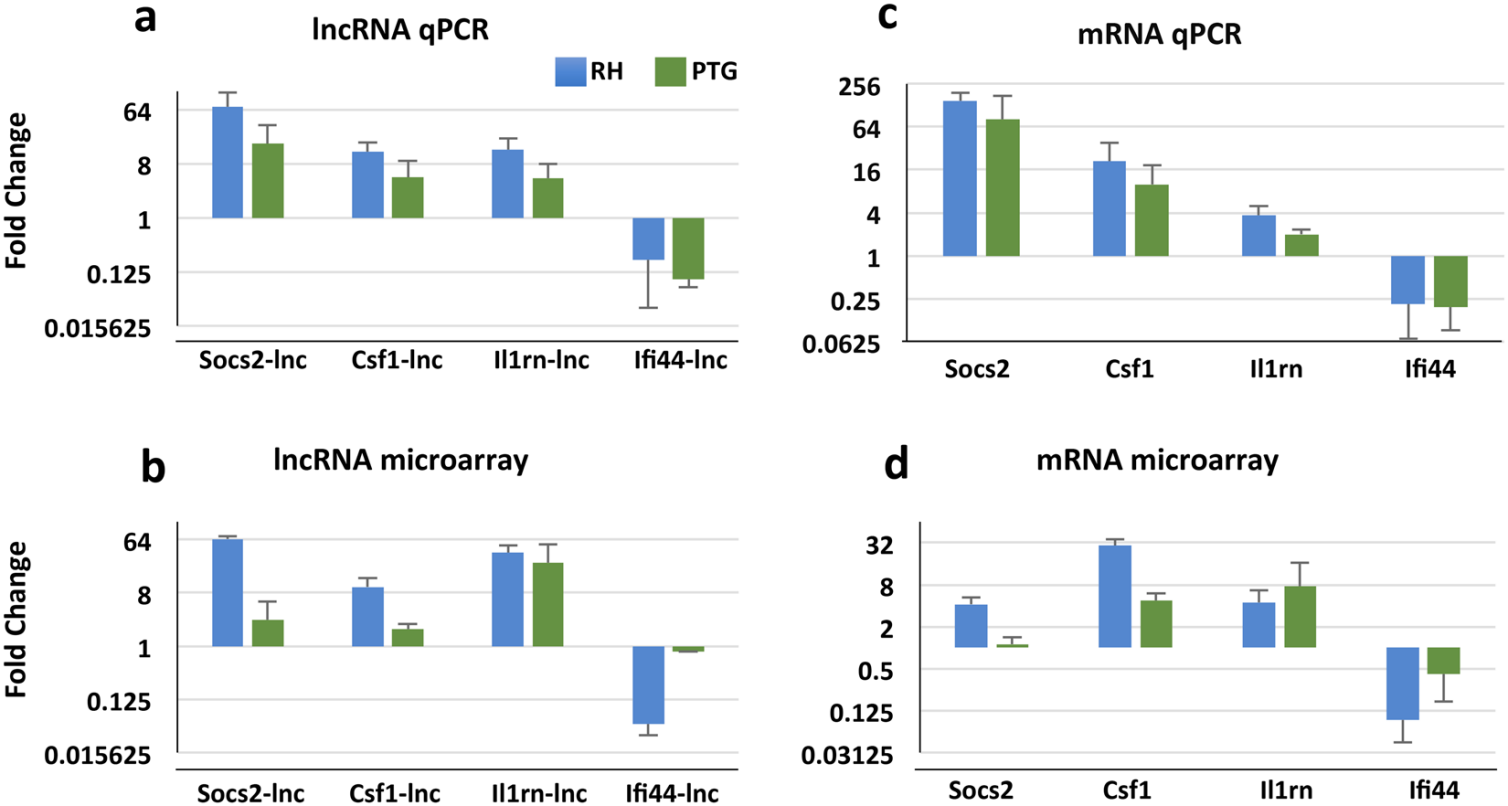
Validation of lncRNA and mRNA microarray data by qRT-PCR. RNA from mouse BMDM were collected 6 hr after infection with either RH or PTG strains of *T*. *gondii*, and qRT-PCR was subsequently performed. Fold changes represent the comparison of infected samples to uninfected samples. Regulation of representative lncRNAs as determined by qPCR (**a**) and microarray (**b**) analysis. Regulation of immune-related mRNA is shown by qPCR (**c**) and microarray (**d**) analysis. Microarray fold change values differ slightly from those listed in Supplementary Spreadsheets 1 and 2 (which are the geometric means) because arithmetic means were calculated from the microarray data to directly compare to qRT-PCR data. Experiments were completed a minimum of three times with BMDM from three separate mice and were obtained independently of experiments used for microarray analysis.

We designed primers to bind a unique region of each lncRNA and co-regulated mRNA. qRT-PCR largely confirmed that lncRNA transcripts were up- or down-regulated (≥or ≤2-fold) during infection with either the RH or PTG in a manner similar to the microarray (Fig. 3a and b). The microarray was considered valid if the directionality (up, down, or not regulated) of transcription changes compared to uninfected samples was consistent between the qRT-PCR data and the microarray data. Likewise, mRNA transcripts were similarly regulated in both the microarray and qRT-PCR (Fig. 3c and d). Nevertheless, during infection with the PTG strain, we identified two loci, Ifi44-lnc and Socs2, that were not expressed in the microarray but did appear to be regulated in the qRT-PCR.

### *Toxoplasma* rhoptry kinase ROP16 manipulates expression of host lncRNA

During intracellular infection, *T*. *gondii* secretes a subset of effector molecules whose activities modify host responses to infection^32^. One example is the rhoptry protein ROP16, a kinase that directly activates host cell STAT3 and STAT6^17,21,33,34^. While Type I ROP16 possesses strong STAT kinase activity, Type II ROP16 is catalytically inactive. Previous studies in BMDM indicate that 538 protein-coding genes are regulated by Type I (RH) ROP16^18^.

**Figure 4:**
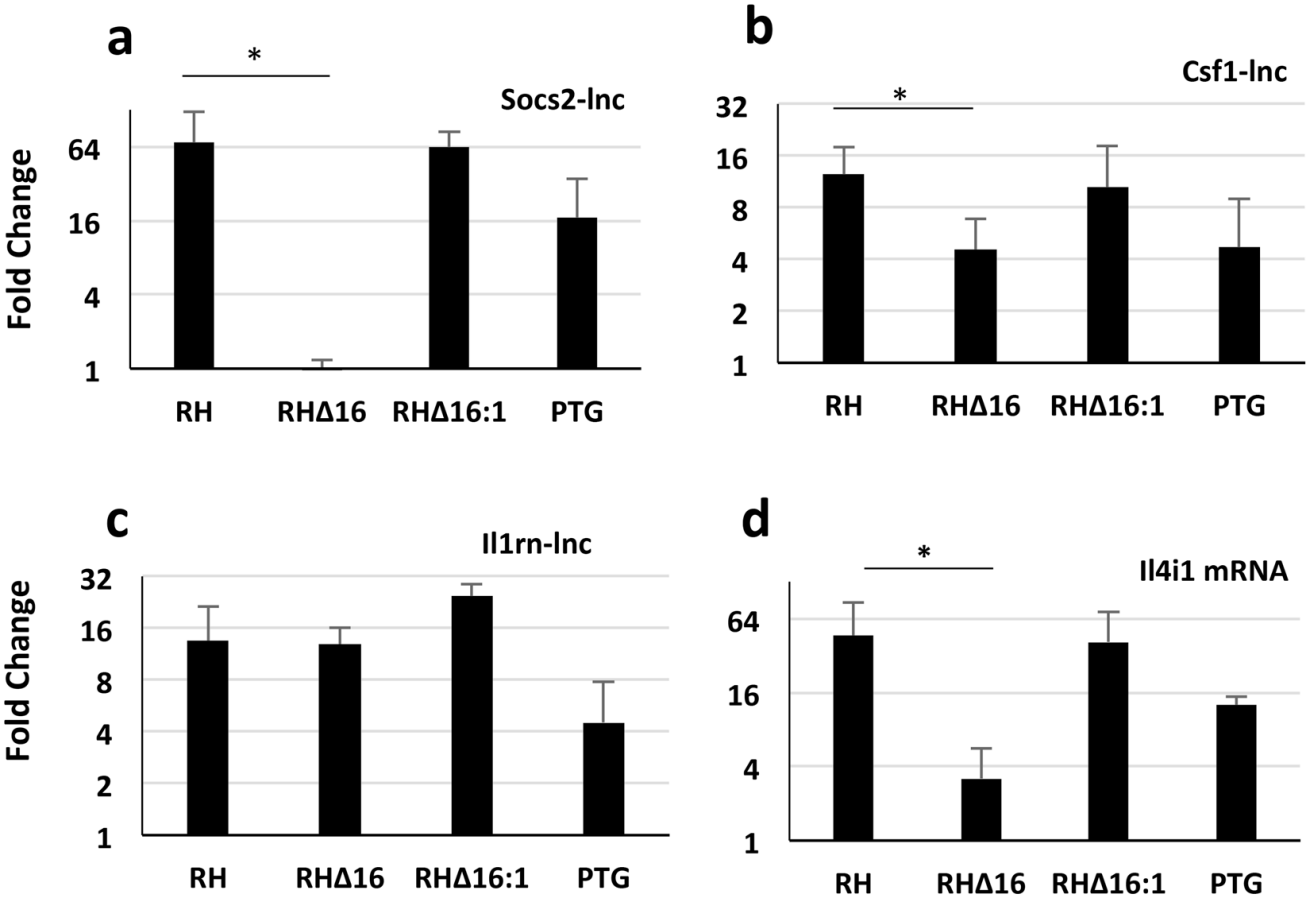
lncRNAs are targeted for manipulation by *T*. *gondii* rhoptry kinase ROP16. BMDM were infected with RH, a ROP16 deletion mutant on the RH background (RHΔ16), a ROP16 complementation strain (RHΔ16:1), and PTG. Then, RNA was isolated 6 hr later for qRT-PCR analysis. Expression patterns of Socs2-lnc (**a**), Csf1-lnc (**b**), Il1rn-lnc (**c**) and Il4i1 mRNA (**d**) were determined. Fold changes represent the comparison of infected samples to uninfected samples. *p* < 0.05 was considered significant when comparing RH and RH∆16. The experiments were completed a minimum of three independent times.

We hypothesized that the activity of ROP16 extends to control of lncRNAs, particularly those strongly up-regulated by the Type I RH strain relative to the Type II PTG. By using engineered ROP16 deletion and complementation mutants created on the RH background^17^, we examined expression of three lncRNAs that showed evidence of Type I strain-specific up-regulation: Socs2-lnc, Csf1-lnc, and Il1rn-lnc. Expression of Csf1-lnc and Socs2-lnc was significantly reduced during infection with the ROP16 deletion mutant (RH∆16) compared to RH (Fig. 4a and b). Complementation of RH∆16 with a functional copy of ROP16 (RH∆16:1) restored the up-regulation phenotype. In contrast, Il1rn-lnc was not controlled by ROP16, and the expression of Il1rn-lnc RH∆16 in Fig. 4c is similar to that of RH and RH∆16:1. As a positive control for a gene controlled by ROP16, we examined the expression of an mRNA (Il4i1) that we suspected was also controlled by ROP16 based upon the strain-specific expression pattern. Il4i1 protein-coding mRNA expression was also significantly lower in the RH∆16 strain (Fig. 4d). Our data demonstrate that the secreted parasite effector molecule ROP16 regulates several putative host lncRNAs.

Additionally, to isolate effects of live infection from other potential sources of gene regulation (such as the presence of dead parasites or soluble parasite protein), we examined expression of Socs2-lnc after addition of live, dead, and soluble tachyzoite antigen (STAg). Addition of dead parasites and STAg did not result in increased expression of Socs2-lnc, suggesting that Socs2-lnc is induced by live *T*. *gondii* infection (Supplementary Fig. S5).

## Discussion

In this study we employed a microarray approach to comprehensively survey host lncRNAs that are regulated during intracellular infection of macrophages with *T*. *gondii*. We identified ~900 putative lncRNAs that were up-regulated by infection, and a similar number whose expression was down-regulated. A subset of the parasite-regulated lncRNAs was associated with mRNA transcripts that encode immune response genes. We selected four of these lncRNA molecules for validation by qRT-PCR: Socs2-lnc, Csf1-lnc, Il1rn-lnc, and Ifi44-lnc. We determined that expression of Socs2-lnc and Csf1-lnc depended on the parasite rhoptry kinase ROP16. Our lncRNA screen was performed *in vitro* using a single host cell type, and therefore it is possible that we are missing some important lncRNAs that are induced *in vivo* but not in our BMDM cultures. Nevertheless, the fact that we have identified several lncRNAs that are regulated by *Toxoplasma* in our study of macrophages (which are a major host reservoir during *in vivo* infection) provides rich ground for further analysis. We also cannot rule out that some of our observed expression effects may not be due to live infection and could be due to the presence of dead parasites, the presence of soluble parasite extract, or the phagocytosis of live parasites. While some of our identified lncRNAs may be induced by these other factors, they still may be important for the immune response to *T*. *gondii* infection.

The microarray simultaneously enabled us to examine the expression of 24,881 mRNAs, and we identified 1,927 protein-coding transcripts that were differentially regulated during *T*. *gondii* infection. Our results are consistent with prior studies on BMDM responses to *Toxoplasma*. For example, expression levels of ccl17, csf1, ccl24, ccl7, cxcl2, and Socs2 in response to infection agree with previous publications^18,35^. While the focus of the present study was on lncRNA expression patterns, the mRNA expression analysis allows the placement of this study in the context of previous work.

We examined lncRNA responses to infections with Type I RH and Type II PTG parasite strains, which are commonly found in European and North American host populations. The Type I strains exhibit a highly virulent phenotype in mice in that one tachyzoite is sufficient to cause death before encystment^36–38^. There is also epidemiological evidence that Type I strains cause more severe pathology in humans^39,40^. The Type II strains display decreased virulence in mice and can establish long-term infection through formation of quiescent cysts. Previous experiments using BMDM show that Type I tachyzoites drive an M2 phenotype, whereas Type II parasites drive an M1 phenotype. Therefore, some of the strain-specific effects on lncRNA expression may reflect generalized M1/M2 responses^17,18^. Infection with Type I RH induces a massive pro-inflammatory cytokine response that likely contributes to death^41,42^. Paradoxically, *in vitro* studies show that Type II strains elicit stronger IL-12 responses from macrophages, a response that results from NFκB activation by the parasite secretory molecule GRA15^43–45^. It is also well known that *Toxoplasma* Type I strains evade host IFN-γ-induced immunity-related GTPase destruction of the parasitophorous vacuole through the activities of secretory rhoptry proteins ROP5, ROP17 and ROP18^46–50^. The findings in the present study indicate that the impact of parasite strain type also extends to host lncRNA expression. For example, we observed a substantial difference in the number of differentially regulated lncRNAs between Type I RH and Type II PTG (1522 and 528, respectively). Given the emerging understanding of the importance of lncRNAs in infection and immunity^3^, we hypothesize that noncoding RNA responses will prove to be important determinants of virulence.

During invasion, tachyzoites inject the rhoptry protein ROP16 into the host cell cytoplasm. Type I ROP16 is a host-directed kinase that phosphorylates STAT3 and STAT6. Type II parasite strains express a form of ROP16 that lacks kinase activity. In addition to cytoplasmic STAT activation, ROP16 contains a nuclear localization sequence^21^. This characteristic raises the possibility that ROP16 possesses other activities that may require localization to the host cell nucleus. In this regard, a yeast 2 hybrid system was recently used to identify host proteins Dnaja1 (DnaJ heat shock protein family member A1) and Gabra4 (gamma-aminobutyric acid A receptor, subunit alpha 4) as non-STAT proteins that interact with ROP16^51^.

Here, we identify two lncRNA molecules whose expression depends upon ROP16, namely Csf1-lnc and Socs2-lnc. Csf1-lnc is a lncRNA associated with the gene that encodes the cytokine Csf1, which drives hematopoietic stem differentiation into macrophage lineage cells. Socs2-lnc is a lncRNA that overlaps with the Socs2 gene. Socs2 has previously been implicated in anti-inflammatory responses to *T*. *gondii*^28^. In the case of Socs2-lnc, deletion of the entire ROP16 gene on the Type I background had a much more profound impact than loss of ROP16 kinase activity in Type II PTG. One explanation of this result is that on a Type II genetic background the dense granule protein GRA15 controls Socs2-lnc, as has been shown for the Socs2 gene itself^18^. Another explanation of this result is that Socs2-lnc expression is controlled by kinase domain-independent portions of the ROP16 protein.

The role of lncRNA in the immune system, particularly in the context of infection, is not well understood, but its importance is becoming increasingly clear^3,52,53^. During viral infection, for example, lncRNAs NEAT1, lncRNA-CMPK2, and lncBST2 control production of IFN and other antiviral cytokines^54–56^. Other lncRNAs, including GAS5, interfere with assembly by direct binding to viral structural components^57^. In mouse macrophages, both stimulation with bacterial lipopolysaccharide and infection with *Listeria monocytogenes* induces expression of lncRNA AS-IL1α and intergenic lncRNAs lincRNA-Cox2 and lincRNA-Tnfaip3. Through distinct mechanisms, each of these noncoding RNAs regulates expression of pro-inflammatory cytokines^6,58,59^.

While our work was in progress, another group used a similar microarray approach to identify lncRNA expression in human foreskin fibroblast cells infected with only Type II *T*. *gondii*^60^. In that study, a lncRNA designated NONSHAT022487 was up-regulated during infection of macrophages. Based on correlation analysis, it was suggested to play a role in suppression of UNC93B1, a molecule involved in TLR11/12-mediated recognition of *Toxoplasma*^61,62^. Our study extends this work by showing that parasite strain exerts a strong influence on lncRNA expression patterns. We also show that the secretory kinase ROP16 controls expression of a subset of lncRNAs that are linked to immune response genes. Given the abundance of lncRNAs encoded within the genome as well as the increasing number of studies indicating their regulatory function, we hypothesize that lncRNAs are major regulators of the host response to *Toxoplasma* and other microbial pathogens.

## Materials and Methods

### Parasites and infections

Wildtype *Toxoplasma* strains RH and PTG, as well as engineered strains RH∆16 and RH∆16:1, were used in this study. The latter two strains were constructed and kindly provided by D. Bzik and B. Fox (Dartmouth Geisel School of Medicine). The construction of both mutant strains has been described previously^17^. In brief, the ROP16 coding region was deleted in the Type I RH strain KU80 knockout background by homologous recombination of a HXGPRT marker. The RH∆16 strain was then complemented with a single copy of a functional allele of ROP16 from Type I RH by replacement of HXGPRT at the ROP16-deleted locus to generate strain RH∆16:1. RH was heat-treated for 30 minutes at 65 °C to kill *T*.*gondii* for experiments with inactivated parasites. Soluble tachyzoite antigen (STAg) was prepared according to a previously published protocol^63^. STAg was added to BMDM at a concentration of 20 µg per 10^6^ BMDM.

Tachyzoites of all strains were maintained by approximately twice-weekly passage on human foreskin fibroblast monolayers in human fibroblast medium (HFM) consisting of DMEM (Life Technologies) supplemented with 1% heat-inactivated bovine growth serum (Thermo Fisher Scientific), 100 U/mL penicillin (Life Technologies), and 0.1 mg/mL streptomycin (Life Technologies). Infections were accomplished by addition of tachyzoites to mouse BMDM (4:1 ratio of parasites to cells) on 12-well plates (Falcon, non-tissue culture treated). Plates were briefly centrifuged (3 min, 200 × g) to initiate contact between tachyzoites and macrophages. Cultures were incubated 6 hrs (37 °C, 5% CO_2_), then cells were harvested for RNA extraction. Percent infection and number of tachyzoites per infected cell were calculated using an Olympus BX51 immunofluorescence microscope. DAPI was used to stain the nucleus. *Toxoplasma*-specific polyclonal antibody conjugated to FITC (ThermoFisher Scientific) was used to stain the parasites.

### Generation of BMDM

Femur and tibia from female C57BL/6 mice (6–8 wks of age; The Jackson Laboratory) were used as a source of bone marrow cells. Macrophages were generated from single cell suspensions of bone marrow cells by 5-day culture in L929-containing supernatants, as previously described^64^. One day prior to infection, BMDM were harvested, counted, and plated on 12-well tissue culture plates in HFM at a concentration of 1–2 × 10^6^ cells per well.

### Microarray and data analysis

Total RNA was prepared from BMDM by RNeasy Mini Kit purification (Qiagen). Total RNA from each sample was quantified using a NanoDrop ND-1000 (ThermoFisher Scientific), and the RNA integrity was assessed using standard denaturing agarose gel electrophoresis. Microarray analysis was carried out by Arraystar, Inc. using the Mouse LncRNA v3.0, an Agilent Array platform (Agilent Technologies, Inc.). The sample preparation and microarray hybridization were performed based on Agilent standard protocols with minor modifications. Briefly, mRNA was purified from total RNA after removal of rRNA (mRNA-ONLY™ Eukaryotic mRNA Isolation Kit, Epicentre). Then, each sample was amplified and transcribed into fluorescent cRNA along the entire length of the transcripts without 3′ bias utilizing a mixture of oligo(dT) and random primers (Arraystar Flash RNA Labeling Kit, Arraystar). The labeled cRNAs were hybridized onto the Mouse LncRNA Array v3.0 (8 × 60 K, Arraystar). After washing slides, the arrays were scanned by the Agilent Scanner G2505C.

Agilent Feature Extraction software (version 11.0.1.1) was used to analyze the acquired array images. Quantile normalization and subsequent data processing were performed using the GeneSpring GX v12.1 software package (Agilent Technologies). After quantile normalization of the raw data, lncRNA and mRNA for which at least 3 out of 9 samples had flags in Present or Marginal (“All Targets Value”) were chosen for further data analysis. Differentially expressed lncRNA and mRNA with statistical significance were identified through Volcano Plot filtering between two groups using the R software package. Pathway analysis and GO analysis were applied to determine the roles that these differentially expressed mRNA played in these biological pathways or GO terms (https://www.genome.jp/kegg/; http://www.geneontology.org/). Hierarchical clustering, using R heatmap.2, was performed to display the distinguishable lncRNA and mRNA expression patterns among samples. Normalized intensities were used as the values. The distance metric was Euclidean and the linkage criterion was average. (https://www.rdocumentation.org/packages/gplots/versions/3.0.1/topics/heatmap.2) (https://www.rdocumentation.org/packages/stats/versions/3.5.1/topics/hclust)

Additional data analysis was performed using the Microsoft Excel software.

### Quantitative RT-PCR

Total RNA was prepared using a RNeasy Mini Kit (Qiagen), and samples were subjected to Turbo DNase treatment (Life Technologies). RNA was converted to cDNA using the SuperScript IV VILO Master Mix (ThermoFisher Scientific). Quantitative PCR was performed on target genes and normalized to the expression of the housekeeping gene *Ppia* using the SYBR green method (SsoAdvanced Universal SYBR Green Supermix, Bio-Rad) and the Bio-Rad CFX96 RT-PCR machine. Expression relative to uninfected control samples was calculated using the ∆∆C_t_ method. A control with no added reverse transcriptase was included for each sample. Primer sequences used are shown in Table 4. For our Socs2-lnc primers, some of our samples (such as uninfected and RHΔ16) were occasionally at the limit detection of our qRT-PCR assay. In those cases, we used 35 cycles as the Cq.

**Table 4:** Primer sequences used for qRT-PCR analysis.

Il4i1 mRNA	F	CAACAGGGAAAGGGGCCATTC
	R	GGCCTTGAGGTCTTTGAAGGC
Ppia mRNA	F	GCATGTGGTCTTTGGGAAGGTG
	R	GGGTAAAATGCCCGCAAGTCAA
Ifi44 mRNA	F	CCAGGGGAAAAGCACACAAAACA
	R	TGCGATTGGTCCTTGTAGCTGA
Csf1 mRNA	F	TTGCTAAGTGCTCTAGCCGAGG
	R	AGGTGGAAGACAGACTCAGGGA
Il1rn mRNA	F	GCAACCACCTTGAGCCTGAAAT
	R	TTAGGTGACTGTAGGGTCCCCA
Socs2 mRNA	F	CAAGATCCCTTGTGCCCGGAG
	R	GCAGAATGGTGTGGCAAAGTCT
Csf1 lncRNA	F	CGGTAGTGATGGAGTGTGGCTT
	R	CTGCCTGTACCTCTGGATTGCT
Socs2 lncRNA	F	AGATGGCTCAGTTACGTGCCTT
	R	ACCTGACCAATTCTGCTCAGCT
Il1rn lncRNA	F	TGTGTGCCTTACAGGGTGAACA
	R	TTGATGGCATCTCCCAAGGCTT
Ifi44 lncRNA	F	GGTGGGCTGTGATGAAGATGGA
	R	AAGTTGAACCGAGAAGCCTGGG
uc.70 lncRNA	F	ATCAGGGAGAGATCTCAGGCCA
	R	CCCCTGGCCTTCTGTGAGAATG

Primers were designed to encompass unique regions of each lncRNA and mRNA examined.

### Statistical Analyses

Microarray expression results from Arraystar were transformed from logarithmic values to linear values. This step enabled the direct comparison of arithmetic means and standard deviations of the microarray and qRT-PCR data. Error bars in Figs 3 and 4 are shown as the standard deviation of 3–6 biological experiments. Grubbs’ test, with a significance level of 0.05, was used to determine if a replicate was an outlier and discard it. Dixon’s *Q* test gave comparable results. Expression differences in lncRNAs and in mRNAs were tested for significance using an unpaired Student t-test. Two tailed tests were performed, with *p* < 0.05 considered statistically significant.

### Ethical Approval

All experimental protocols were approved by the University of New Mexico Institutional Animal Care and Use Committee (IACUC). The methods were carried out in accordance with the Panel on Euthanasia of the American Veterinary Medical Association.

## Electronic supplementary material

Dataset 1

Dataset 2

Dataset 4

Dataset 3

Dataset 5

Dataset 6

## Data Availability

All data generated or analyzed during this study are included in this published article (and Supplementary Information files).
