# Supplementary material for: Toxoplasma gondii Manipulates Expression of Host Long Noncoding RNA during Intracellular Infection
Source: Sci Rep. 2018 Oct 9;8:15017. doi: 10.1038/s41598-018-33274-5 (PMC6177471; doi:10.1038/s41598-018-33274-5)
Supplement: Supplementary file 1 — Dataset 1 [file 41598_2018_33274_MOESM1_ESM.pdf]

Toxoplasma gondii Manipulates Expression of Host Long Noncoding RNA during Intracellular Infection

Kayla L. Menard, Breanne E. Haskins, Anthony P. Colombo,
and Eric Y. Denkers

Supplementary Figures 1-5

Supplementary Legends

Supplementary Figure S1. Efficiency of infection by RH and PTG tachyzoites. BMDM were infected with the indicated parasite strains at a 4:1 MOI, then 6 hr later cells were collected and stained with FITC-labeled anti-*Toxoplasma* antibody (ThermoFisher) and DAPI. In some experiments cells were subjected to Diff-Quick staining. (**a**) Percent infection over n=7 (RH) and n=5 (PTG) independent experiments. (**b**) Average number of tachyzoites (TZ) per infected cell (n=90 per strain). ns, non-significant. (**c**) Representative immunofluorescence images of infected BMDM.

Supplementary Figure S2. Validation of ultraconserved region uc.70 by qRT-PCR. RNA from mouse BMDM was collected 6 hr after infection with either RH or PTG strains of *T. gondii*, and qRT-PCR was subsequently performed. Fold changes represent the comparison of infected samples to uninfected samples. Microarray fold change values differ slightly from those listed in Supplementary Spreadsheets 1 and 2 (which are the geometric means) because arithmetic means were calculated from the microarray data to directly compare to qRT-PCR data. Experiments were completed a minimum of three times with BMDM from three separate mice and were obtained independently of experiments used for microarray analysis.

Supplementary Figure S3. Gene ontology (GO) analysis demonstrates that many infection and immune-related biological processes were significantly enriched among the differentially expressed mRNAs. The top biological process enrichment scores for each comparison are shown. (**a**) up-regulated RH vs. uninfected, (**b**) down-regulated RH vs. uninfected, (**c**) up-regulated PTG vs. uninfected, (**d**) down-regulated PTG vs. uninfected, (**e**) up-regulated PTG vs. RH, and (**f**) down-regulated PTG vs. RH (http://www.geneontology.org/).

Supplementary Figure S4. KEGG Pathway analysis of mRNA showing significant enrichment of infection and immune-related pathways among the differentially expressed mRNAs. The top pathway enrichment scores for each comparison are shown. (**a**) up-regulated RH vs. uninfected, (**b**) down-regulated RH vs. uninfected, (**c**) up-regulated PTG vs. uninfected, (**d**) down-regulated PTG vs. uninfected, (**e**) up-regulated PTG vs. RH, and (**f**) down-regulated PTG vs. RH (https://www.genome.jp/kegg/).

Supplementary Figure S5. Live tachyzoites, but not heat-killed or soluble tachyzoite extract, trigger lncRNA-Socs2 expression. BMDM were incubated with live RH strain tachyzoites (MOI 4:1), heat-killed tachyzoites (4:1 ratio of parasites to cells) or STAg (16.7 µg/ml, corresponding to the equivalent number of tachzoites). After 6 hr, cells were harvested and RNA was prepared for qPCR. The results shown are values averaged for 2 biological replicates.

Supplementary Data S1. Complete list of differentially regulated lncRNAs. Each tab in the spreadsheet corresponds to a specific pairwise comparison (e.g. RH vs. uninfected) and direction of fold change (up or down). Fold changes, source, raw microarray values, and other information are included.

Supplementary Data S2. lncRNAs differentially regulated during both RH and PTG infection. Each tab corresponds to a specific combination: up-regulated in RH and PTG, down-regulated in RH and PTG, up-regulated in RH and down-regulated in PTG, down-regulated in RH and up-regulated in PTG.

Supplementary Data S3. Complete list of differentially regulated mRNAs**.** Each tab in the spreadsheet corresponds to a pairwise comparison (e.g. RH vs. uninfected) and direction of fold change (up or down).

Supplementary Data S4. mRNAs differentially regulated during both RH and PTG infection. Each tab in the spreadsheet corresponds to a specific combination: up-regulated in RH and PTG, down-regulated in RH and PTG, up-regulated in RH and down-regulated in PTG, down-regulated in RH and up-regulated in PTG.

Supplementary Data S5. lncRNAs co-regulated with an associated (adjacent or overlapping) mRNA. Each tab in the spreadsheet corresponds to a pairwise comparison (e.g. lncRNA up-regulated and associated mRNA up-regulated). Some comparisons (e.g. PTG lncRNA up-regulated and mRNA down-regulated) did not have any co-regulated lncRNAs and mRNAs. In this case, "NONE" is listed in the tab. Known or suspected immune- and infection-related mRNAs are highlighted in orange.

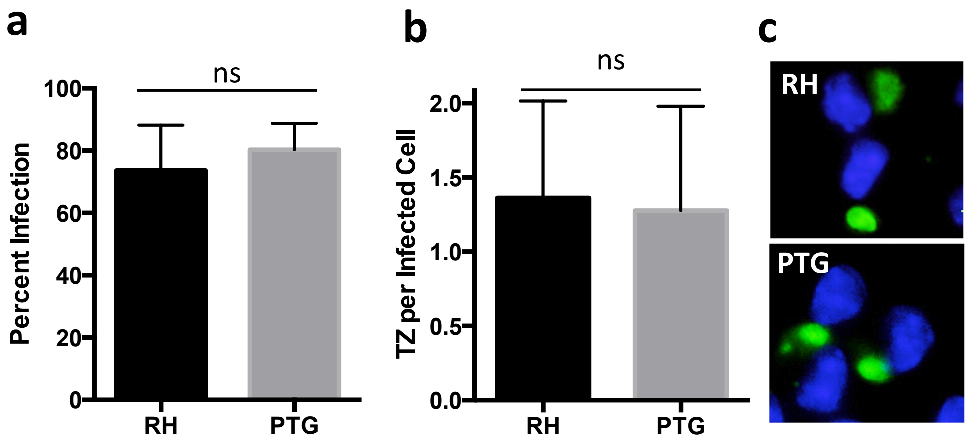

Supplementary Figure-S1 (Denkers)

Supplementary Figure S1. Efficiency of infection by RH and PTG tachyzoites. BMDM were infected with the indicated parasite strains at a 4: 1 MOI, then 6 hr later cells were collected and stained with FITC-labeled anti-*Toxoplasma* antibody (ThermoFisher) and DAPI. In some experiments cells were subjected to Diff-Quik staining. (**a**) Percent infection over n=7 (RH) and n=5 (PTG) independent experiments. (**b**) Average number of tachyzoites (TZ) per infected cell (n=90 per strain). ns, non-significant. (**c**) Representative immunofluorescence images of infected BMDM.

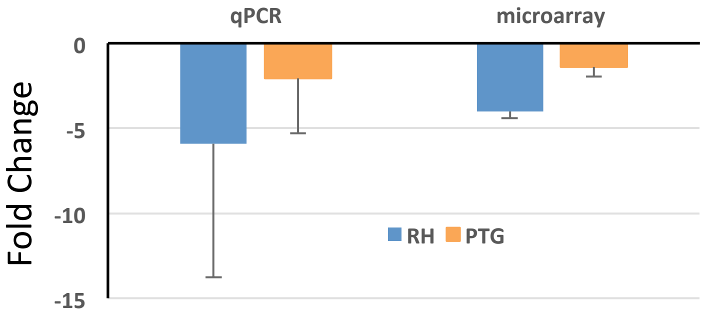

Supplementary Figure-S2 (Denkers)

Supplementary Figure S2. Validation of ultraconserved region uc.70 by qRT-PCR. RNA from mouse BMDM was collected 6 hr after infection with either RH or PTG strains of *T. gondii*, and qRT-PCR was subsequently performed. Fold changes represent the comparison of infected samples to uninfected samples. Microarray fold change values differ slightly from those listed in Supplementary Spreadsheets 1 and 2 (which are the geometric means) because arithmetic means were calculated from the microarray data to directly compare to qRT-PCR data. Experiments were completed a minimum of three times with BMDM from three separate mice and were obtained independently of experiments used for microarray analysis.

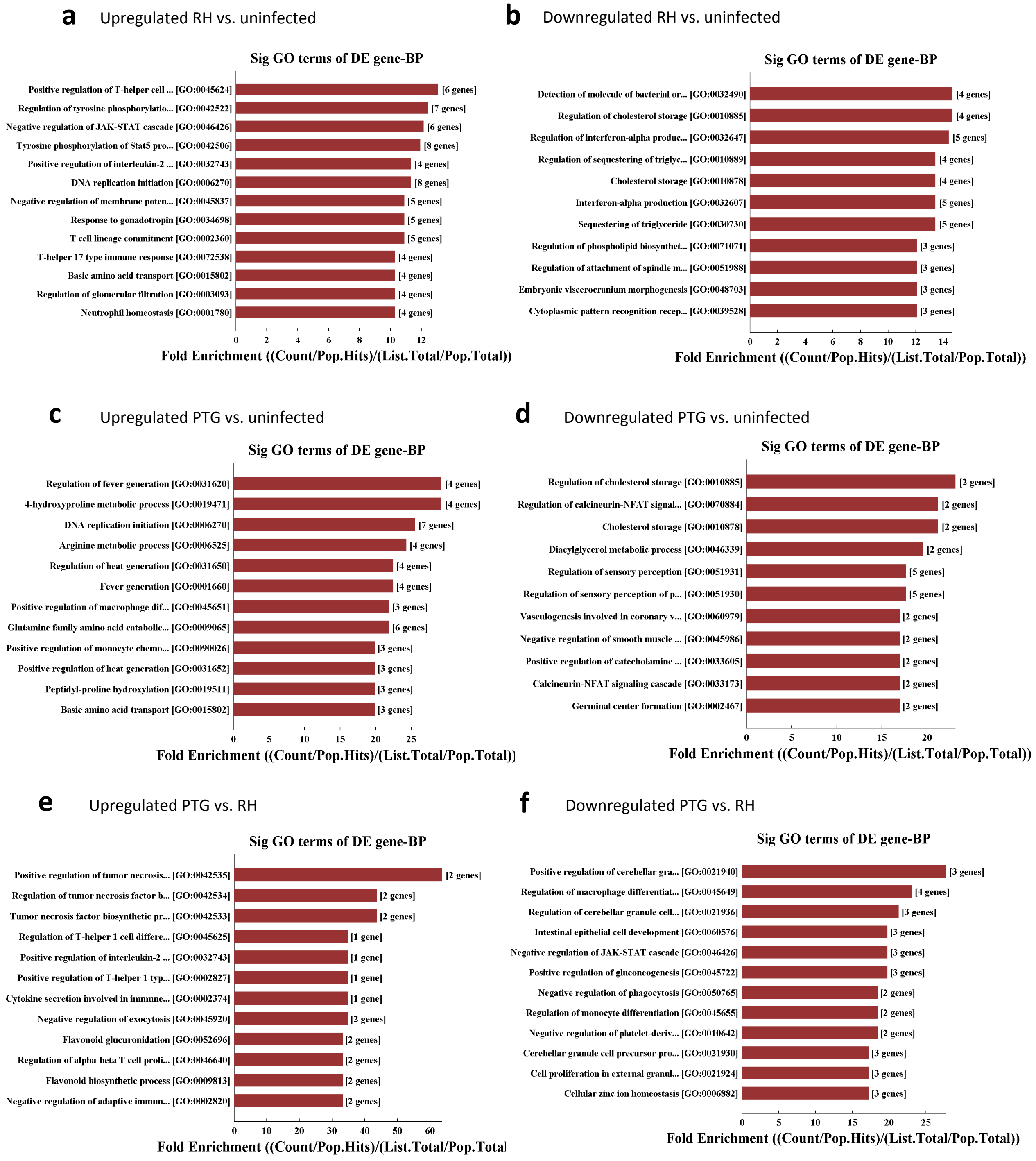

Supplementary Figure-S3 (Denkers)

Supplementary Figure S3. Gene ontology (GO) analysis demonstrates that many infection and immune-related biological processes were significantly enriched among the differentially expressed mRNAs. The top biological process enrichment scores for each comparison are shown. (**a**) upregulated RH vs. uninfected, (**b**) downregulated RH vs. uninfected, (**c**) upregulated PTG vs. uninfected, (**d**) downregulated PTG vs. uninfected, (**e**) upregulated PTG vs. RH, and (**f**) upregulated PTG vs. RH (http://www.geneontology.org/)

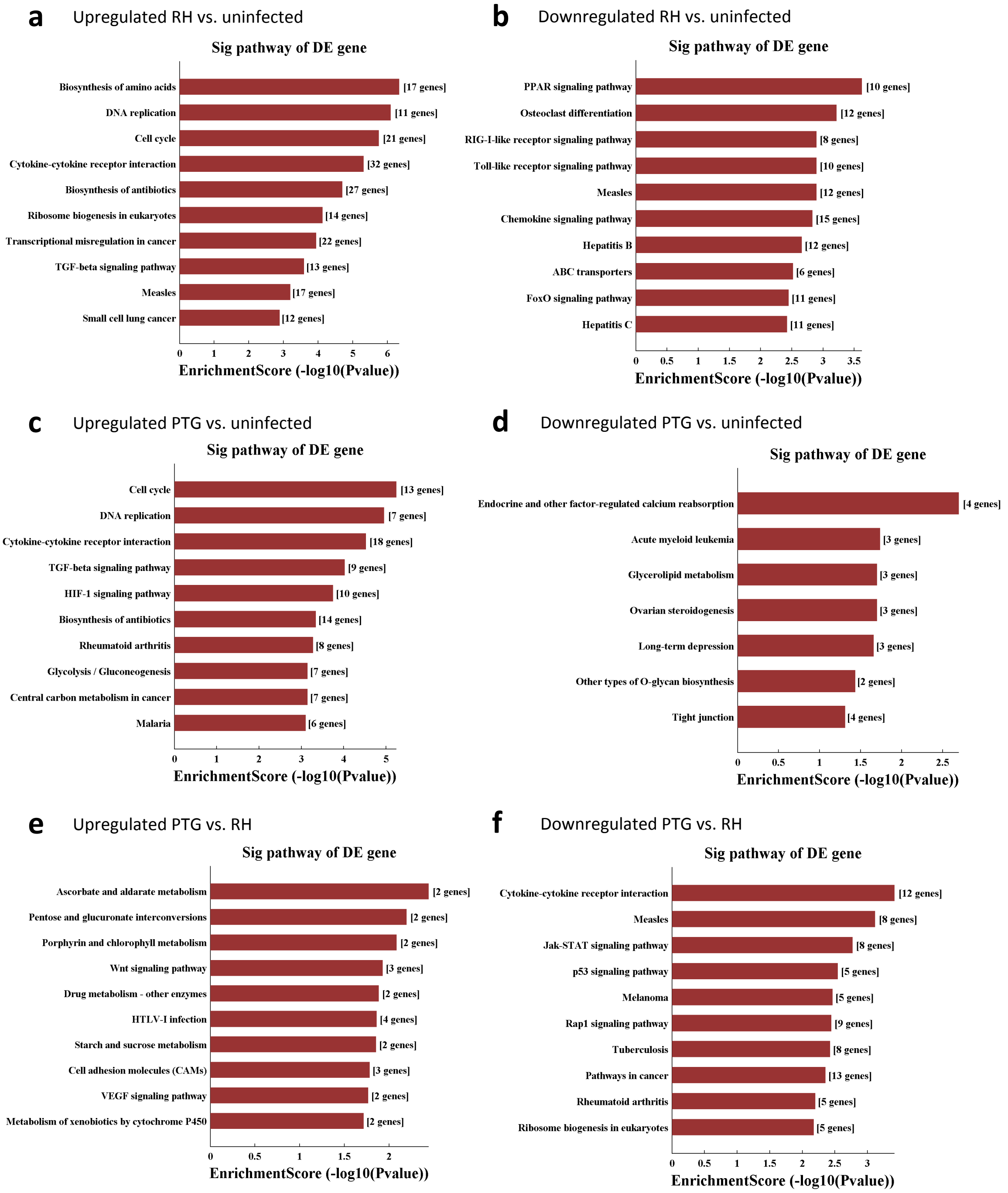

Supplementary Figure-S4 (Denkers)

Supplementary Figure S4. KEGG Pathway analysis of mRNA showing significant enrichment of infection and immune-related pathways among the differentially expressed mRNAs. The top pathway enrichment scores for each comparison are shown. (**a**) upregulated RH vs. uninfected, (**b**) downregulated RH vs. uninfected, (**c**) upregulated PTG vs. uninfected, (**d**) downregulated PTG vs. uninfected, (**e**) upregulated PTG vs. RH, and (**f**) upregulated PTG vs. RH (https://www.genome.jp/kegg/)

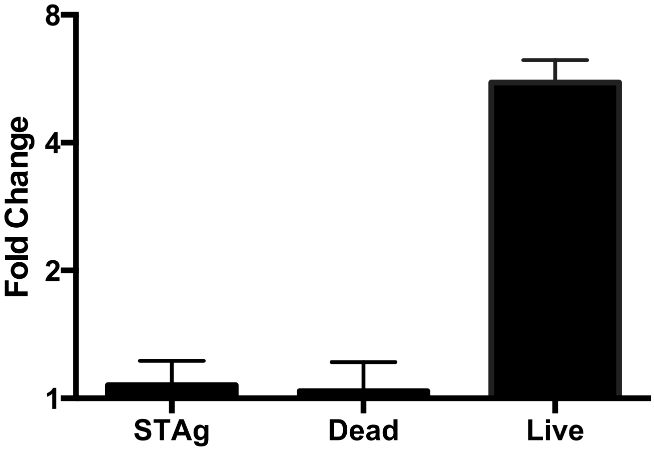

Supplementary Figure-S5 (Denkers)

Supplementary Figure S5. Live tachyzoites, but not heat-killed or soluble tachyzoite extract, trigger lncRNA-Socs2 expression. BMDM were incubated with live RH strain tachyzoites (MOI 4:1), heat-killed tachyzoites (4:1 ratio of parasites to cells) or STAg (16.7 µg/ml, corresponding to the equivalent number of tachzoites). After 6 hr, cells were harvested and RNA was prepared for qPCR. The results shown are values averaged for 2 biological replicates.
